# Effect of Gender on Patients with Non-Small-Cell Lung Cancer Treated with Immune Checkpoint Inhibitors: A Real-World Study

**DOI:** 10.3390/biomedicines13020437

**Published:** 2025-02-11

**Authors:** Teresa Calleja-Chucla, Patricia Cordeiro González, Alejandro Martínez Pradeda, Sonia Pértega-Díaz, Luis Margusino-Framiñán, Silvia Antolín Novoa

**Affiliations:** 1 Servicio de Farmacia, Complejo Hospitalario Universitario A Coruña, 15006 A Coruña, Spain; luis.margusino.framinan@sergas.es; 2 Departamento de Ciencias de la Salud, Universidade da Coruña (UDC), 15006 A Coruña, Spain; alejandro.martinez@cog.es; 3 Grupo de investigación de Farmacia Hospitalaria, Instituto de Investigación Biomédica de A Coruña (INIBIC), Complexo Hospitalario Universitario de A Coruña (CHUAC), Sergas, Universidade da Coruña (UDC), 15006 A Coruña, Spain; 4 Instituto de Investigación Biomédica de A Coruña (INIBIC), 15006 A Coruña, Spain; patricia.cordeiro.gonzalez@sergas.es; 5 Servicio de Oncología Médica, Complejo Hospitalario Universitario A Coruña, 15006 A Coruña, Spain; 6 Servicio de Farmacia, Centro Oncológico de Galicia, 15009 A Coruña, Spain; 7 Rheumatology and Health Research Group, Department of Health Sciences, Faculty of Nursing and Podiatry, Esteiro, Universidade da Coruña, 15403 Ferrol, Spain; 8 Nursing and Health Care Research Group, Instituto de Investigación Biomédica de A Coruña (INIBIC), Xubias de Arriba 84, 15006 A Coruña, Spain

**Keywords:** sex, immune checkpoint inhibitor, non-small-cell lung cancer

## Abstract

**Objective:** To evaluate the differences in overall survival (OS) and progression-free survival (PFS) between men and women with non-small-cell lung cancer (NSCLC) treated with immune checkpoint inhibitors (ICIs) in second-line and later treatments. **Methods:** A retrospective, single-center observational study was conducted on patients with advanced NSCLC treated with ICIs (nivolumab, pembrolizumab, and atezolizumab) from January 2015 to December 2019 (with follow-up until December 2021). Demographic, clinical, and treatment-related variables were collected. OSand PFSwere analyzed using the Kaplan–Meier method and compared between genders using the log-rank test.A multivariate Cox regression analysis was performed to adjust for confounders. **Results:** A total of 189 patients were included, and 47 (25%) were women. The most common histology was adenocarcinoma (61%). Women began treatment at a younger age (59.8 vs. 66 years, *p* < 0.001) and had higher rates of active smoking (46.8% vs. 38%, *p* = 0.001). The median OS was similar between men (9.5 months, 95% CI: 7.1–11.8) and women (9.2 months, 95% CI: 3.3–15.2; *p* = 0.382) while PFS was significantly higher in males (3.2 months, 95% CI: 2.5–4.0) than in females (2.1 months; 95% CI = 1.6–2.5) (*p* = 0.002).Women had higher rates of tumor cachexia (BMI < 20).Worse PFS was observed for women both in the <20 kg/m^2^ (median PFS: 1.8 vs. 2.7 months, *p* = 0.016) and 20–24.9 kg/m^2^ groups (median PFS: 2.2 vs. 3.3 months, *p* = 0.077), while in patients with a BMI >= 25 kg/m^2^, median OS was higher in women than in men (14.7 months vs. 10.1 months). Women had also a significantly worse PFS than men among those with a cumulative tobacco consumption of <30 packs-year (median PFS: 2.2 vs. 3.2, *p* = 0.038. In the multivariate analysis, the male sex was significantly associated with a better PFS(HR = 0.59; *p* = 0.009), without significant differences between sexes in OS (HR = 0.90; *p* = 0.618). Among the other variables analyzed, only an ECOG >= 2 was significantly associated with both worse OS (HR = 3.53; 95% CI = 1.93–6.47) and PFS (HR = 2.19; 95% CI = 1.23–3.89). Women who discontinued due to toxicity (*n* = 7) had a median OS of 41.4 months (95% CI: 14.7–68.1) after discontinuation, whereas men (*n* = 15) had a median OS of 8.8 months (95% CI: 6.9–10.8), (*p* = 0.045). **Conclusions:** Sex-based differences were observed in the ICI outcomes. Women had worse PFS, particularly with lower BMI and lower tobacco exposure, despite similar OS between sexes. Women discontinued ICIs due to toxicity earlier but showed longer OS after discontinuation. Poor ECOG status was linked to worse outcomes across all the patients.

## 1. Introduction

Lung cancer (LC) currently represents one of the greatest healthcare challenges due to its rising incidence. In 2022, LC was the most diagnosed cancer worldwide, with nearly 2.5 million new cases, accounting for 12.4% of all global cancers, followed by breast cancer (11.6%), colorectal cancer (9.6%), prostate cancer (7.3%), and stomach cancer (4.9%) [1]. In Spain, according to the Spanish Society of Medical Oncology (SEOM) and the Spanish Network of Cancer Registries (REDECAN), there were 22,316 new cases reported in men and 8632 in women in 2022 [2]. These findings are alarming, especially considering that 90% of the LC cases are attributable to smoking, a preventable cause [3].

Traditionally, LC has been more common in men than in women. Among men, the implementation of preventive policies and increased awareness of the risks associated with smoking have led to a stabilization in the annual incidence of lung cancer (LC). In some countries, a decline in diagnoses has even been observed compared to the previous years. However, among women, the progressive rise in smoking prevalence has resulted in a continued upward trend in LC incidence, presenting significant challenges for public health systems and oncology care strategies.

Immune checkpoint inhibitors (ICIs) have brought a paradigm shift in the treatment of non-small-cell lung cancer (NSCLC), leading to significant improvements in long-term survival and quality of life for patients [4,5,6,7]. However, a significant proportion of patients fail to respond to this treatment. While the predictors of response to these drugs remain imprecisely understood, substantial evidence underscores the crucial role of the immune system in combating tumor cells [8].

It is known that certain diseases manifest with different frequency, severity, or symptoms depending on sex due to the interactions between endogenous factors (such as genetics, hormones, and the immune system) and environmental factors (such as lifestyle and toxin exposure). This dimorphism is also seen in cancer [9,10,11]. In NSCLC, women have been observed to have a lower risk of cancer-specific mortality [3]. However, when examining the subgroup of patients treated with immunotherapy, this sex disparity diminishes or even reverses. Since 2017, several meta-analyses have investigated whether differences exist in immunotherapy effectiveness between men and women. Most studies have identified differences, suggesting that men may respond better to monotherapy with immunotherapy, although some authors have found no significant sex-based differences. Conversely, some publications indicate that women may have a better response to immunotherapy compared to men (Table 1).

Despite these findings, the same oncology treatments continue to be applied to both sexes, with the clinical trial results often predominantly based on male participants, being extrapolated to the female population, which tends to be underrepresented. Therefore, it is essential to deepen research through sex-specific studies to optimize treatments. This underscores the importance of conducting studies with greater female representation to better understand treatment response differences, thus advancing toward more effective therapies for both sexes, as sex is typically not considered a stratification factor in clinical trials. On the other hand, most evidence on sex-based survival differences in lung cancer patients treated with immunotherapy comes from randomized clinical trials, which, while providing high-quality data, often include highly selected patient populations and operate under controlled conditions that may not fully reflect the complexity of real-world clinical practice. This highlights the need for studies in real-world settings, where the diversity of patient profiles and healthcare delivery conditions can provide a more comprehensive understanding of these associations [12].

**Table 1:** Studies on the influence of gender on the response to immunotherapy.

Reference	Tumor	Type of Drug (ICI)	Efficacy
(2017) **[13]**	NSCLC, melanoma, renal, and urothelial	PD-1 and CTLA-4	Anti PD-1: OS without statistical significance in men: HR 0.72 vs. 0.81, *p* = 0.285. CTLA-4, OS HR 0.65 men vs. 0.79 women, *p* = 0.078.
**[14]**	NSCLC, melanoma, renal, urothelial, gastric, and head and neck	PD-1, PDL-1, and CTLA-4	Similar benefit, slightly higher OS in men: HR 0.73 vs. 0.77, *p* < 0.001. CTLA-4 OS benefit in men: HR 0.77 (*p* = 0.0012) vs. HR 0.89 (*p* = 0.162).
**[15]**	NSCLC andmelanoma	PD-1, PDL-1, and CTLA-4	Similar benefit, slightly greater in men: HR 0.62 vs. 0.74, *p* = 0.373.
**[3]**	NSCLC	PD-1, EGFR, and ALK	Women have a lower risk of cancer-specific death (HR = 0.73; *p* < 0.00001). OS with EGFR is better in women (HR:0.34 vs. HR: 0.44). Similar OS with ALK (HR: 0.51 vs. HR: 0.48; *p* < 0.00001).
**[16]**	NSCLC	PD-1 and PDL-1	A better combination of QT and immunotherapy in women: HR 0.48 vs. 0.76. No benefit of immunotherapy alone in women HR 0.78 vs. 0.97, *p* = 0.002.
**[17]**	NSCLC	PD-1, PDL-1, and CTLA-4	No significant difference in OS: HR 0.75 vs. 0.77, *p* = 0.6.
**[18]**	NSCLC	PD-1 and PDL-1	More consistent and favorable ICI results in male patients than in female patients.
**[19]**	NSCLC	PD-1, PDL-1, and CTLA-4	Greater benefit in men: HR 0.68 vs. 0.82 in women, *p* < 0.05.
**[20]**	NSCLC	PD-1, PDL-1, and CTLA-4	Greater OS benefit in men (squamous) (HR: 0.73, 95% CI 0.58–0.91) vs. women (HR: 0.74, 95% CI 0.37–1.48).
**[21]**	NSCLC	PD-1 and PDL-1	No significant difference in OS in men vs. women: HR 0.74 (95% CI 0.66–0.83) vs. 0.72 (95% CI 0.63–0.82), *p* = 0.709, nor PFS HR 0.63 (95% CI 0.53–0.75) vs. 0.72 (95% CI 0.58–0.88), *p* = 0.372.
(2022) **[22]**	NSCLC	Inhibidor de PD-1 and PDL-1	No significant differences in OS (*p* = 0.054): Greater trend towards better PFS in men vs. women (HR = 5 vs. 4.5, *p* = 0.133).

NSCLC: non-small-cell lung cancer; PD-1: Programmed Cell Death Protein 1; PDL-1: Programmed DeathLigand 1; CTLA-4: Cytotoxic T-Lymphocyte Antigen 4; OS: overall survival; ICIs: immune checkpoint inhibitors; HR: Hazard Ratio; ALK: Anaplastic Lymphoma Kinase.

This study aims to evaluate gender differences in the overall survival (OS) and progression-free survival (PFS) among men and women with NSCLC treated with ICIs in second-line and later therapies.

## 2. Materials and Methods

### 2.1. Study Design

This is a single-center retrospective observational study that included all the patients diagnosed with advanced NSCLC who received at least one dose of ICIs (nivolumab (Bristol-Myers Squibb, New York, NY, USA), pembrolizumab (Merck & Co., Inc., Rahway, NJ, USA), or atezolizumab (Genentech, a member of the Roche Group, South San Francisco, CA, USA)) in second-line or later treatments and were followed-up by the medical oncology department at a tertiary hospital between January 2015 and December 2019. Patient follow-up continued until 31 December 2021. The study excluded patients who were treated with ICIs for other diagnoses and patients under 18 years of age. Data were obtained from the electronic medical records system (IANUS^®^) and electronic prescription system (Farmis_Oncofarm^®^, V4.15.2).

For each participant, the following patient-related variables were collected: sex, metastatic site, general condition at the start of the ici treatment (assessed by Eastern Cooperative Oncology Group (ECOG) performance status), tumor stage at diagnosis based on TNM classification, tumor histology (squamous, adenocarcinoma, or other), PDL-1 status, cumulative tobacco consumption (CT, defined as packs per year), body mass index (BMI), and treatment-related variables—type of ICI, age at treatment initiation, treatment line (2nd or3rd), OS, PFS, and reason for treatment discontinuation. OS was defined as the time from ICI initiation to death from any cause. PFS was defined as the time from ICI initiation to disease progression or death, whichever came first. Disease progression was defined by the Immune Response Evaluation Criteria in Solid Tumors as an increase in the sum of maximum tumor diameters ≥25% relative to nadir, the development of any new lesions, or an unequivocal increase in non-measurable malignant disease.

### 2.2. Statistical Analysis

Statistical analysis was carried out using the SPSS^®^ version 28 and R^®^ version 4.4.2 software packages. First, a descriptive analysis of the variables collected was performed by sex. For qualitative variables, frequency distributions and percentages were calculated. For quantitative variables, the values were expressed as mean ± SD (median). Differences in percentages were evaluated using the chi-square test or Fisher’s exact test, as appropriate. Mean comparisons between the two groups were performed using Student’s *t*-test or Mann–Whitney non-parametric test, after testing for normality using the Kolmogorov–Smirnov test. OS and PFS were determined for both men and women using the Kaplan–Meier method and compared using the log-rank test. Finally, a multivariate Cox regression analysis was performed to identify differences between the male and female groups after adjustment for the other patient and disease characteristics.Bilateral *p*-values were obtained and considered statistically significant if *p* < 0.05.

The study was conducted in accordance with the Declaration of Helsinki and approved by the Drug Research Ethics Committee (CEIm) (protocol code: TCC-ATE-2018-01) (Ethics Committee code: 2018/601 and approved on 28 February 2019) for studies involving humans.

## 3. Results

A total of 189 patients were included, 47 (25%) female and 142 (75%) male, with a mean age of 64.8 (SD: 9.8) years. Patient, disease, and disease characteristics for both sexes are shown in Table 2. The most common histology in both sexes was adenocarcinoma (61% of the cases, 72.3% in women and 57.7% in men, *p* = 0.075). The most common ECOG score at the start of treatment was 1 (64.6%).

The age of treatment initiation was earlier in women (59.8 ± 9.7 years) than in men (66.6 ± 9.3 years) (*p* < 0.001).Women presented at more advanced stages (stage IV at diagnosis in 66% of the women and 57.7% of the men), but this difference was not statistically significant (*p* = 0.320).Among the metastatic patients, there were no differences in bone (10.6% of the women vs. 17.6% of the men), liver (8.5% vs. 10.6%), or other extracerebral metastases. In contrast, the women in our series were more likely to have dissemination to the central nervous system (6.4% vs. 1.4%), although this difference did not reach statistical significance (*p* = 0.099).

The women studied were more likely to be of normal weight (mean BMI 24.0 ± 4.3 kg/m^2^) than overweight, whereas overweight was more common in men (mean BMI 26.5 ± 4.7 kg/m^2^ (*p* = 0.002). Regarding differences in smoking status, more current smokers were observed in women (46.8% vs. 38.0%), whereas more ex-smokers were observed in men (59.9% of the men reached the diagnosis without smoking compared to 36.2% of the women) (*p* < 0.001). The men in our series had a significantly higher cumulative tobacco consumption (57.5 ± 26.4 vs. 34.4 ± 24.6 packs-year; *p* < 0.001).

Only six patientswere still receiving ICIs at the end of the follow-up. The main reasons for discontinuation were disease progression (53.2% of the women vs. 52.1% of the men) or death (23.4% of the women vs. 19.7% of the men), with toxicity being the reason for discontinuation in 14.9% of the women and 10.6% of the men. Treatment discontinuation due to toxicity occurred earlier in the women than in the men who discontinued for the same reason (4.9 ± 5.3 vs. 7.1 ± 6.6 months) (Table 3).

**Figure 1:**
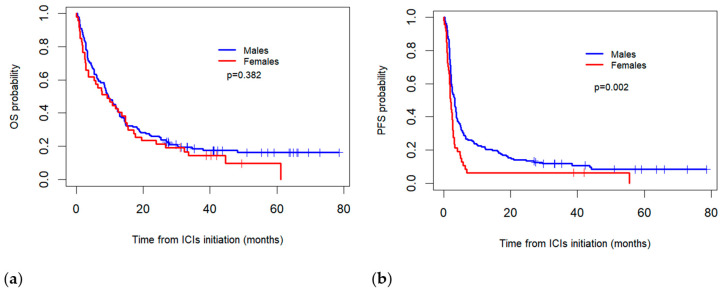
Comparison of Kaplan–Meier overall survival (OS) (**a**) and progression-free survival (PFS) (**b**) curves between sexes for patients treated with immune checkpoint inhibitors (ICIs).

The median real-world OS for the females was 9.2 months (95% CI: 3.3–15.2) compared to 9.5 months (95% CI: 7.1–11.8) for the males, with no statistically significant difference observed between the two groups (*p* = 0.382). Meanwhile, the median PFS was significantly higher in the males (3.2 months, 95% CI: 2.5–4.0) than in the females (2.1 months; 95% CI = 1.6–2.5) (*p* = 0.002) (Figure 1).

**Figure 2:**
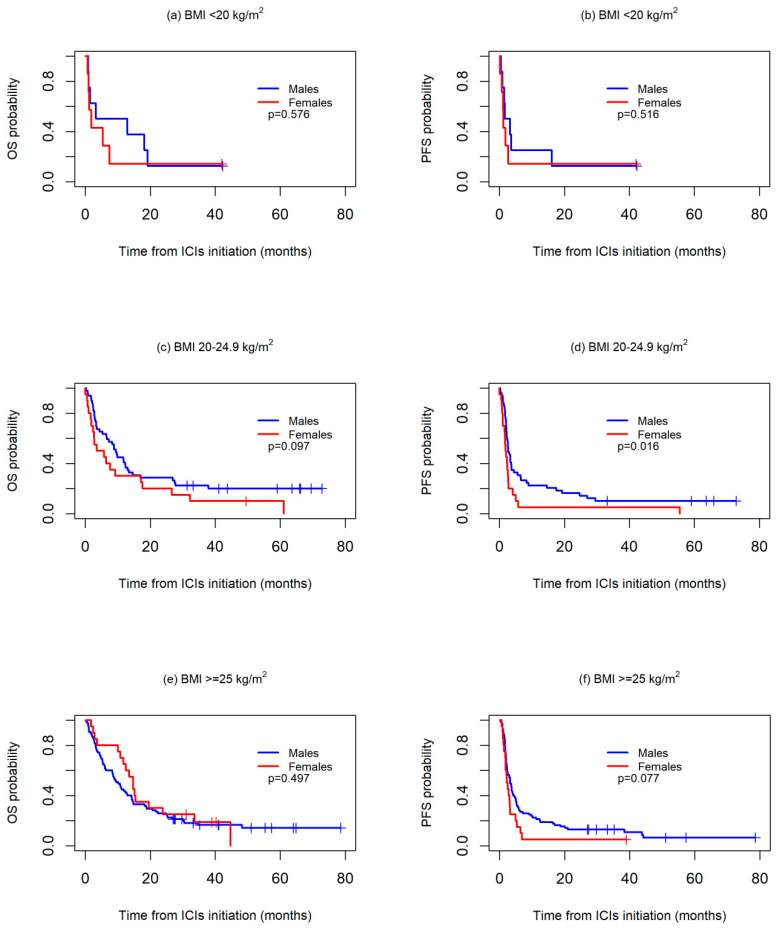
Kaplan-Meier overall survival (OS) (**a**,**c**,**e**) and progression-free survival (PFS) (**b**,**d**,**f**) curves between sexes, according to body mass index (BMI) values.

Differences according to sex were further investigated in the subgroups stratified by BMI and cumulative tobacco consumption since both sexes showed marked differences according to these two variables.Survival was first analyzed across three BMI categories: <20 kg/m^2^, 20–24.9 kg/m^2^, and ≥25 kg/m^2^. No significant differences were observed between the sexes in any of the three groups, although median OS was worse in the women than in the men both in the patients with a BMI <20 kg/m^2^ (1.8 months vs. 3.2 months) or a BMI 20–24.9 kg/m^2^ (3.5 months vs. 9.4 months), while in the patients with a BMI >=25 kg/m^2^, median OS was higher in women than in men (14.7 months vs. 10.1 months).With regard to PFS, no differences were observed between sexes in the BMI <20 kg/m^2^ group (median PFS: 1.2 vs. 1.7 months, *p* = 0.516), whereas a worse PFS was, in general, observed for the women both in the 20–24.9 kg/m^2^ (median PFS: 1.8 vs. 2.7 months, *p* = 0.016) and >=25 kg/m^2^ groups (median PFS: 2.2 vs. 3.3 months, *p* = 0.077) (Figure 2).

**Figure 3:**
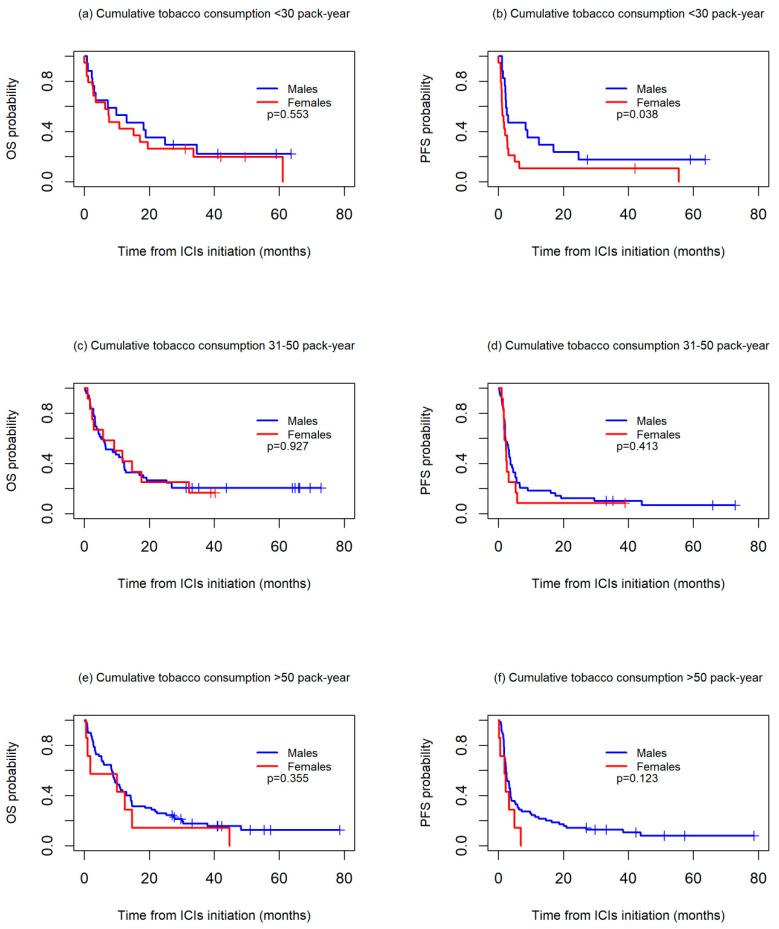
Kaplan-Meier overall survival (OS) (**a**,**c**,**e**) and progression-free survival (PFS) (**b**,**d**,**f**) curves between sexes, according to cumulative tobacco consumption, in packs-years.

OS and PFS worsened in the patients with higher cumulative tobacco consumption in both the men and women. For OS, no differences between sexes were observed in any of the three groups studied, while for PFS, the women had a significantly worse prognosis than the men among those with a cumulative tobacco consumption of <30 packs-year (median PFS: 2.2 vs. 3.2, *p* = 0.038), with differences not reaching statistical significance among those with a cumulative consumption of 30–50 packs-year (median PFS: 1.6 vs. 3.0, *p* = 0.413) or >50 packs-year (median PFS: 2.4 vs. 3.3, *p* = 0.123) (Figure 3).

After adjustment for patient, disease, and treatment variables, differences in PFS according to sex persisted, with the male sex significantly associated with a better prognosis (HR = 0.59; 95% CI = 0.40–0.87). However, the multivariate analysis confirmed that sex was not associated with OS (HR = 0.90; 95% CI = 0.60–1.36). Among the other variables analyzed in this study, only an ECOG performance status >=2 was significantly associated with both worse OS (HR = 3.53; 95% CI = 1.93–6.47) and worse PFS (HR = 2.19; 95% CI = 1.23–3.89) (Table 4).

**Figure 4:**
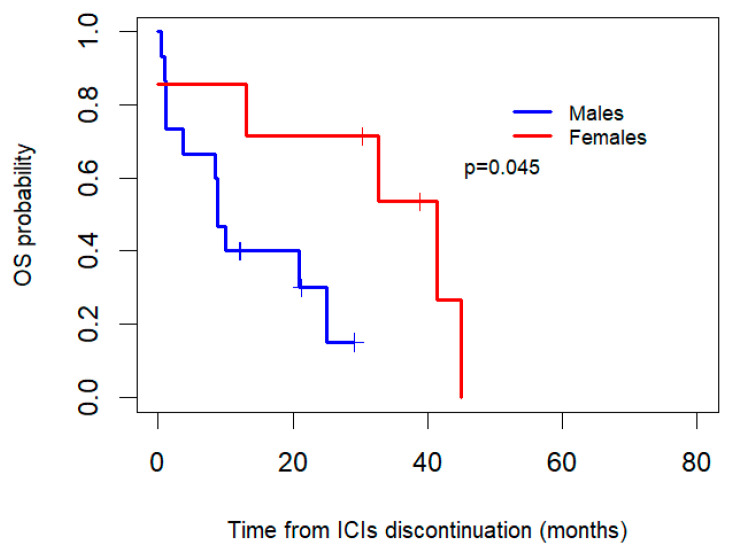
Kaplan–Meier overall survival (OS) curves by gender for patients who discontinued ICIs due to toxicity.

Additionally, OS after the discontinuation of ICIs was compared betweenthe women and men who discontinued due to toxicity (Figure 4). After the interruption of ICIs, the women who discontinued due to toxicity (*n* = 7) had a median OS of 41.4 months (95% CI: 14.7–68.1), whereas the men (*n* = 15) had a median OS of 8.8 months (95% CI: 6.9–10.8), with an associated *p*-value of 0.045.

## 4. Discussion

Sex differences in the efficacy and toxicity of ICIs in LC care is an area of active research. The impact of biological sex (male and female) on the efficacy and toxicity of ICIs has been investigated, as sex may influence tumor biology, immune response, and drug metabolism [19,20,22,23].

In our cohort of metastatic NSCLC patients treated with ICIs in second and subsequent lines, the women were diagnosed at a younger age, had lower smoking rates, and showed a tendency to present at more advanced stages with greater dissemination to the central nervous system.This is consistent with the hypothesis that women are more susceptible to smoking than men [9,24]. Traditionally, men have had higher smoking rates than women, which partly explains their higher incidence of NSCLC. However, in recent years, smoking rates in women have increased or remained more stable, especially in certain age groups, leading to a proportional increase in NSCLC cases in this group. Women appear to be more susceptible to the harm caused by smoking in terms of their risk of developing lung cancer.

We do not know whether these differences can be attributed to a greater deleterious effect of the toxicants contained in tobacco in women, to a more recent incorporation into this toxic addiction, or whether other factors also play a role, such as hormonal environment or passive smoking. In other words, it seems that women might be more sensitive to the harmful effect of tobacco and possibly present a more invasive disease, debuting already with disseminated disease or showing a greater tendency to develop hyperaggressive forms that overcome the blood–brain barrier.

Several meta-analyses have found a worse response of women to PD-1/PD-1L inhibitors [13,16] even without gender differences in the use of conventional chemotherapy and immunotherapy [3]. Other authors found no differences [19,21], or slight differences [14,15]. These meta-analyses mentioned above are based on clinical trials only, and in some cases also include subjects treated with anti-CTLA-4 or targeted therapies (such as EGFR or ALK inhibitors). In our case, we have conducted a real-life study, so we have included all the patients treated with PD-1/PD-1L inhibitors, which could provide more richness in the population characteristics.

NSCLC is the second cancer with the most somatic mutations after melanoma [25], so given that the immune system is more active in women and autoimmune diseases are more common, it would be expected that the effectiveness in this subgroup would be greater [26]. Both our findings and the reported literature indicate that this hypothesis does not hold true, as there appears to be greater effectiveness of these drugs in men than in women. This could be due to the fact that tumors in men tend to be more immunogenic than those in women, perhaps because of longer exposure to different toxins, mainly tobacco [18]. In this context, although the microenvironment in women tends to be more reactive, tumor immunogenicity tends to be lower. Some authors point out that in women, better results are obtained with combinations of chemotherapy and immunotherapy than with immunotherapy alone [16]. It is hypothesized that the greater benefit of the combination in women is due to the fact that chemotherapy releases more tumor antigens, making it possible for the more powerful female immune system to act [13,16].

In this study, OS did not differ significantly between men and women. This finding aligns with previous research suggesting that sex may not always influence OS in some cancer treatments [27]. However, the median progression-free survival (PFS) was significantly lower in females compared to males. This discrepancy in PFS may indicate that women, despite similar OS, experience a quicker progression of disease during treatment. Several factors could contribute to the observed differences in PFS. One possibility is the distinct biological response to cancer treatment between sexes. For instance, women may experience faster tumor progression or differences in the tumor microenvironment that affect treatment efficacy. Hormonal differences, particularly estrogen, may also influence cancer progression and response to therapies, as estrogenis known to affect immune response and tumor behavior.The estrogenic state in women could influence the response to immune ICIs, as estrogens modulate immunity and the tumor microenvironment. For instance, estrogen regulates the expression of PD-L1, a key target of ICIs, and can alter immune cell activity within the tumor environment. This suggests that hormonal levels, such as those associated with the menstrual cycle, menopause, or hormone therapy, might impact the efficacy of ICIs in cancer treatment [28].

It is possible that women in our population have a higher incidence of tumor-associated cachexia compared to men, with low weight in women serving as a more significant negative prognostic factor due to tumor-related malnutrition. Although there are no studies specifically analyzing the effect of BMI by gender, several meta-analyses suggest that overweight and obese NSCLC patients tend to experience longer survival with ICI regimens. This could be attributed to a variety of factors, including better nutritional status or the metabolic effects of excess weight that may influence the efficacy of treatment. Our study supports the growing evidence that cachexia is a critical prognostic and predictive factor for the effectiveness of ICIs.

The findings of this study suggest that higher cumulative tobacco consumption is associated with worse PFS in both men and women, consistent with the previous literature that highlights the detrimental impact of smoking on cancer outcomes. However, a notable difference was observed between the sexes: the women with lower cumulative tobacco consumption (<30 packs-year) had significantly worse PFS compared to the men, while no such differences were seen in the individuals with higher levels of tobacco consumption. These findings suggest that the effects of smoking on cancer progression may be more pronounced in women at lower levels of tobacco exposure, potentially indicating sex-specific differences in the biology of tobacco-related cancers. Several studies have documented that smoking can influence the efficacy of cancer treatments, including immune ICIs [29,30,31,32,33,34].

It has been postulated in the literature that women experience more adverse events (AEs) secondary to chemotherapy, and recent studies also indicate a greater tendency to experience more AEs with immunotherapy. This phenomenon could be explained by the immune hyperactivity discussed throughout this text [35]. Importantly, the women who discontinued treatment due to toxicity might have shown a trend towards longer OS compared to the men, which could be due to the factors mentioned above.To the best of our knowledge, no evidence or studies have been found to address this specific hypothesis.

In terms of limitations, a major concern with our study is its single-center design, which may limit the generalizability of the results. In addition, the study does not include data from the most recent patient populations treated with combination chemotherapy and immunotherapy in both first-line and advanced settings, as these cohorts require longer follow-up to obtain mature survival results. On the other hand, the exhaustive follow-up of the included patients minimizes the risk of information bias despite the retrospective nature of the study, which relies on data collected from medical records. It should also be noted that potential confounding bias may persist because adjustments were not made for all the possible variables; however, key confounders essential for the accurate assessment of sex differences were carefully adjusted in the analysis.

## 5. Conclusions

This study highlights notable sex-based differences in outcomes among patientstreated with ICIs. While OS did not differ significantly between sexes, the women experienced consistently worse PFS compared to the men, particularly in the subgroups with lower BMI orlower cumulative tobacco consumption. Despite similar rates of treatment discontinuation due to disease progression, death, or toxicity, the women discontinued ICIs due to toxicity earlier than the men. Interestingly, the women who discontinued treatment due to toxicity demonstrated a significantly longer OS compared to the men in the same group, suggesting a potential differential impact of treatment interruption based on sex.

Multivariate analysis confirmed that the male sex was associated with a better prognosis for PFS, while sex was not significantly associated with OS after adjustment for the other variables. Additionally, poor ECOG performance status emerged as a strong predictor of worse OS and PFS, emphasizing the role of baseline functional status in patient outcomes.

These findings underline the importance of incorporating sex-specific considerations into treatment planning, patient monitoring, and prognostic assessments for patients receiving ICIs, particularly given the differences in treatment tolerance and post-discontinuation survival. Further research is warranted to explore the underlying mechanisms driving these disparities and to optimize individualized treatment approaches.

## Figures and Tables

**Table 2:** Comparison of patient, disease, and treatment characteristics by sex.

		Females *n* = 47 (24.9%)	Males *n* = 142 (75.1%)	*p*
**Patient-related variables**	**Age at ICI initiation (years), mean ± SD**	59.8 ± 9.7 (58.4)	66.6 ± 9.3 (67.4)	<0.001
	**ECOG Performance Status Scale, *n* (%)**			
	0	12 (25.5%)	31 (21.8%)	0.444
	1	27 (57.4%)	95 (66.9%)	
	>=2	8 (17.0%)	16 (11.3%)	
	**Histology, *n* (%)**			0.075 *
	Adenocarcinoma	34 (72.3%)	82 (57.7%)	
	Squamouscellcardinoma	11 (23.4%)	51 (35.9%)	
	Other	9 (6.3%)	2 (4.3%)	
	**Stage at diagnosis, *n* (%)**			0.320 **
	IA-IIB	4 (8.5%)	11 (7.7%)	
	IIIA	6 (12.8%)	24 (16.9%)	
	IIIB	5 (10.6%)	20 (14.1%)	
	IIIC	1 (2.1%)	5 (3.5%)	
	IVA	22 (46.8%)	49 (34.5%)	
	IVB	9 (19.1%)	33 (23.2%)	
	**Metastatic localization, *n* (%)**			
	Bone	5 (10.6%)	25 (17.6%)	0.257
	Hepatic	4 (8.5%)	15 (10.6%)	0.787
	Suprarenal	1 (2.1%)	9 (6.3%)	0.456
	Renal	2 (1.3%)	2(1.4%)	0.258
	Brain	3(6.4%)	2(1.4%)	0.099
	**Tobacco use, *n* (%)**			<0.001 ***
	Never smoker	8 (17.0%)	3 (2.1%)	
	Current smoker	22 (46.8%)	54(38.0%)	
	Former smoker	17 (36.2%)	85(59.9%)	0.001
	**Cumulative tobacco consumption (packs/year), mean ± SD (median)**	34.4 ± 24.6 (30.5)	57.5 ± 26.4 (51.0)	<0.001
	**PDL-1 positive, *n* (%)**	24 (89%)	68 (81%)	0.396
	**BMI at ICI initiation (kg/m^2^), mean ± SD (median)**	24.0 ± 4.3 (23.1)	26.5 ± 4.7 (25.9)	0.002
	<20	7(15.0%)	8 (5.6%)	
	20–24.9	20 (42.5%)	49 (34.5%)	
	≥25	20 (42.5%)	85 (59.9%)	
**Treatment-related variables**	**ICI received, *n* (%)**			0.169
	Pembrolizumab	12 (25.5%)	20 (14.1%)	
	Nivolumab	29 (61.7%)	96 (67.6%)	
	Atezolizumab	6 (12.8%)	26 (18.3%)	
	**Treatment line, *n* (%)**			0.986
	2nd line	40 (85.1%)	121 (85.2%)	
	3rd line and successive	7 (14.9%)	21 (14.8%)	
	**End of the treatment due to toxicity, *n* (%)**	7 (14.9%)	15 (10.6%)	0.422

BMI: body mass index; ECOG: Eastern Cooperative Oncology Group; ICIs: immune checkpoint inhibitors; PDL-1: Programmed cell death ligand-1; SD: Standard Deviation; * *p*-value comparing the percentage of adenocarcinoma histology; ** *p*-value comparing metastatic disease; *** *p*-value comparing neversmokers.

**Table 3:** Causes of immune checkpoint inhibitor (ICI) discontinuation by sex.

	Males		Females	
Cause of ICI Discontinuation	*n* (%)	Months Treated with ICIs. Mean ± SD (Median)	*n* (%)	Months Treated with ICIs, Mean ± SD (Median)
Ongoing	6 (4.2%)	36.9 ± 4.8 (35.2)	0 (0.0%)	-
Diseaseprogression	74 (52.1%)	5.2 ± 6.6 (2.7)	25 (53.2%)	2.5 ± 1.6 (1.9)
Death	28 (19.7%)	4.1 ± 5.7 (2.0)	11 (23.4%)	1.7 ± 1.5 (1.2)
Toxicity	15 (10.6%)	7.1 ± 6.6 (4.3)	7 (14.9%)	4.9 ± 5.3 (3.2)
Clinicaldeterioration	12 (8.5%)	3.5 ± 4.3 (2.2)	3 (6.4%)	3.0 ± 1.9 (2.4)
2-yearcompletiontreatment	4 (2.8%)	26.1 ± 3.3 (24.8)	1 (2.1%)	25.5
Dropout	1 (0.7%)	16.4	0 (0.0%)	-

ICIs: immune checkpoint inhibitors; SD: Standard Deviation.

**Table 4:** Multivariate Cox regression analysis for overall survival (OS) and progression-free survival (PFS).

	Overall Survival				Progression-Free Survival			
	*p*	HR	95% CI (HR)		*p*	HR	95% CI (HR)	
Sex (male vs. female)	0.618	0.90	0.60	1.36	0.009	0.59	0.40	0.87
Age at ICI initiation, years	0.533	1.01	0.99	1.03	0.487	1.01	0.99	1.02
Non-adenocarcinoma histology	0.121	0.76	0.53	1.08	0.992	1.00	0.72	1.39
ECOG PerformaceStatusscale	<0.001				0.024			
ECOG 1 vs. ECOG 0	0.127	1.36	0.92	2.02	0.351	1.20	0.82	1.75
ECOG >= 2 vs. ECOG 0	<0.001	3.53	1.93	6.47	0.008	2.19	1.23	3.89
BMI (kg/m^2^)	0.211	0.98	0.95	1.01	0.434	0.99	0.96	1.02
Neversmoker	0.748	0.89	0.43	1.82	0.618	0.84	0.43	1.66
Stage IV at diagnosis	0.449	1.14	0.81	1.59	0.305	1.18	0.86	1.63
Treatment line (3rd or successive vs. 2nd line)	0.340	0.80	0.50	1.27	0.399	0.83	0.54	1.28

BMI: body mass index; ICIs: immune checkpoint inhibitors; HR: Hazard Ratio; CI: confidence interval.

## Data Availability

The data supporting the findings of this study are stored at the Hospital Universitario de A Coruña but are not publicly available due to privacy and ethical restrictions. However, they are available from the authors upon reasonable request and with permission from the hospital’s Ethics Committee.

## Abbreviations

BMI	body mass index
CI	confidence interval
ECOG	Eastern Cooperative Oncology Group
HR	Hazard Ratio
OS	overall survival
PFS	progression-free survival
ICI	immune checkpoint
NSCLC	non small lung cell cancer
LC	lung cancer
PD-1	Programmed Death-1
PD-1L	Programmed Death-Ligand 1
